# Trends in myocarditis incidence, complications and mortality in Sweden from 2000 to 2014

**DOI:** 10.1038/s41598-022-05951-z

**Published:** 2022-02-02

**Authors:** Michael Fu, Silvana Kontogeorgos, Erik Thunström, Tatiana Zverkova Sandström, Christian Kroon, Entela Bollano, Maria Schaufelberger, Annika Rosengren

**Affiliations:** 1 Department of Molecular and Clinical Medicine, Institute of Medicine, Sahlgrenska Academy, University of Gothenburg, Gothenburg, Sweden; 2 Sahlgrenska University Hospital VG-Region, Gothenburg, Sweden

**Keywords:** Cardiology, Diseases, Medical research

## Abstract

Investigate trends in myocarditis incidence and prognosis in Sweden during 2000–2014. Little data exist concerning population-trends in incidence of hospitalizations for myocarditis and subsequent prognosis. Linking Swedish National Patient and Cause of Death Registers, we identified individuals ≥ 16 years with first-time diagnosis of myocarditis during 2000–2014. Reference population, matched for age and birth year (n = 16,622) was selected from Swedish Total Population Register. Among the 8 679 cases (75% men, 64% < 50 years), incidence rate/100,000 inhabitants rose from 6.3 to 8.6 per 100,000, mostly in men and those < 50 years. Incident heart failure/dilated cardiomyopathy occurred in 6.2% within 1 year after index hospitalization and in 10.2% during 2000–2014, predominantly in those ≥ 50 years (12.1% within 1 year, 20.8% during 2000–2014). In all 8.1% died within 1 year, 0.9% (< 50 years) and 20.8% (≥ 50 years). Hazard ratios (adjusted for age, sex) for 1-year mortality comparing cases and controls were 4.00 (95% confidence interval 1.37–11.70), 4.48 (2.57–7.82), 4.57 (3.31–6.31) and 3.93 (3.39–4.57) for individuals aged < 30, 30 to < 50, 50 to < 70, and ≥ 70 years, respectively. The incidence of myocarditis during 2000–2014 increased, predominantly in men < 50 years. One-year mortality was low, but fourfold higher compared with reference population.

## Introduction

Myocarditis is an inflammatory disorder of the heart muscle, with either focal or diffuse involvement, and with different causes, clinical presentation and outcome^1^. Compared with other heart conditions, myocarditis is uncommon in Western populations, but also potentially frequently underdiagnosed. Community-acquired myocarditis is considered to be predominantly of viral etiology, although confirmatory proof of this is often difficult to obtain^1^. No comprehensive data on the incidence of myocarditis in the population exist, but the proportion of myocarditis in various clinical populations has been reported^1,2^. For example, myocarditis was reported in 20% of sudden cardiac deaths in young military recruits^3^. In addition, it was one of the most common specific causes in 1278 patients referred to the Johns Hopkins Hospital with dilated cardiomyopathy (DCM)^4^.

Likewise, prognosis in myocarditis is poorly understood, especially for long-term survival and later development of other cardiac disorders such as cardiomyopathy or heart failure (HF). Biopsy-proven myocarditis is associated with a poor prognosis in up to 30% of the patients^1–10^, whereas mild forms of myocarditis are, in many patients, believed to resolve spontaneously^11^.

Most data with respect to myocarditis is derived from specialized centers, or fatal cases where cases will not be representative of all cases diagnosed with myocarditis^2–4^. Sweden has a universal healthcare system that provides low-cost hospital care to all Swedish residents, with all hospitals reporting discharge diagnoses to the Swedish National Inpatient Register and all causes of death reported to the National Death Register.

In full recognition of the many difficulties and limitations inherent in the use of administrative data for this diagnosis, we identified all patients with a discharge diagnosis of acute myocarditis from 2000 to 2014 in an attempt to estimate trends in incidence and prognosis of myocarditis in Sweden.

## Methods

### Swedish National Patient Register and National Cause of Death Register

Registration of hospital discharge diagnoses and causes of death, coded in accordance with the International Classification of Diseases (ICD) system, is mandatory in Sweden, with national coverage of all discharge diagnoses since 1987. All individuals aged ≥ 16 years diagnosed with or who died from myocarditis (ICD codes I40 and I51.4) from 2000 to 2014 were identified from the Swedish National Patient Register (NPR) and the Cause of Death Register (NDR), and paired with two controls without myocarditis, matched for age and sex, from the Swedish Population Register. The NPR was used to retrieve information about diagnoses before, during, and after, the index hospitalization. The individuals were considered as being a part of the younger segment if under 50 years.

Data from the NPR and the NDR for cases and controls are linked to personal identity numbers, unique to each Swedish resident. This provides the possibility to combine registries and get exact data on prior disease, comorbidities and cause of death. We estimated 1-year incidence and mortality through the NPR and the NDR and compared these estimates to controls. Data for the whole population of Sweden were used to obtain standardized mortality rates.

### Review of hospital records

Electronic case records of all hospitalized cases with a discharge diagnosis of myocarditis according to the ICD-10 between 2000 and 2014 at the Sahlgrenska University Hospital, Gothenburg, Sweden, were retrieved. Two physicians reviewed the medical journals to verify the diagnosis of myocarditis at baseline. The diagnosis was considered to be correct if the diagnosis made by the attending physician was supported by typical symptoms and elevated biomarkers. In the absence of this clinical information, usually because the patient had been referred for specialized care at the tertiary care center at the Sahlgrenska University Hospital, a biopsy verified myocarditis or imaging verified myocarditis (or both), were accepted. In total, 507 cases (78.5% men) were systematically reviewed out of whom 421 (83.0%) could be verified as acute myocarditis. The Swedish Ethical Review Authority approved the study (Dnr: 026-16 and T826-17), and the study complies with the Declaration of Helsinki. The informed consent of the participants was waived by the Swedish Ethical Review Board because the data was coded, thus anonymous.

### Statistical analysis

All statistical analyses and data management were performed using SAS software version 9.3 (SAS Institute, Cary, NC, USA). Graphics were drawn in SAS 9.3 and R 3.1.3 (RStudio, Boston, MA, USA)^12^. Differences in baseline characteristics between the study groups were evaluated by the t test as well as the Wilcoxon nonparametric test for continuous variables and the chi-square test for dichotomous data. Standardized rates of incidence of myocarditis were estimated per 100,000 individuals and mortality rates per 1000 person-years. Trends in incidence and mortality were determined by linear regression. The general difference in rates was tested as the point estimate under the assumption of the Poisson distribution. The Cochran-Armitage trend test verified changes in the proportion of deaths due to cardiovascular disease (CVD) during the study period. Variation in follow-up times was assessed with Kaplan–Meier survival analysis. A Cox proportional hazard model adjusted for age, sex and period of the study, and the log-rank test were used to compare hazard ratios (HRs). The significance level was set at p < 0.05.

The odds ratio for having a verified myocarditis case was calculated for year of hospitalization, age and sex. The univariate logistic regression showed that the probability of being assigned a valid diagnosis increased by 15% (95% confidence interval [CI] 1.09–1.22) for each year since hospitalization, decreased by 8% with each year of age (95% CI 0.91–0.94) and was twice as high for men (95% CI 1.25–3.70). The sex difference disappeared when an interaction term of age and sex was introduced into the model along with sex. The overall frequency of valid diagnoses was 421 of 507. The incidence rates per 100,000 were then adjusted under the assumption that the number of valid diagnoses in the NPR followed the same distribution as in the reviewed subset.

## Results

### Population characteristics, incidence rates and trend in 2000–2014

From the patient and death registers, we identified 8679 individuals hospitalized with a first-time diagnosis of myocarditis between 2000 and 2014 in Sweden, with a reference population of 16,622 matched for age and sex without myocarditis. Among patients with myocarditis, 74.8% were men and 63.7% were aged < 50 years. Before the index hospitalization for myocarditis, 7.6% had a prior diagnostic code of acute myocardial infarction, 10.2% HF or DCM, 3.4% pericarditis, 9.2% atrial fibrillation and 30.0% any prior diagnosis of CVD. The corresponding figures for the controls were 3.0%, 0.5%, 0%, 2.2% and 12.3%, respectively (Table 1).

**Table 1:** Baseline data and comorbidities before the index hospitalization or death.

	Myocarditis	Reference population	p-value
Age < 50 years	5524 (63.7)	11,009 (66.2)	
Women	2181 (25.1)	4044 (24.3)	
**Year**			
2000–2004	2560 (29.5)	4783 (28.8)	
2005–2009	2754 (31.7)	5297 (31.9)	
2010–2014	3365 (38.8)	6542 (39.4)	
Cancer (all forms)	518 (6.0)	658 (4.0)	< 0.0001
Hypertension	876 (10.1)	800 (4.8)	< 0.0001
Diabetes	485 (5.6)	410 (2.5)	< 0.0001
Myocardial infarction	659 (7.6)	318 (1.9)	< 0.0001
Any coronary heart disease	1074 (12.4)	655 (3.9)	< 0.0001
Any cardiovascular disease	2604 (30.0)	2046 (12.3)	< 0.0001
Heart failure/dilated cardiomyopathy	882 (10.2)	81 (0.5)	< 0.0001
Acute pericarditis	291 (3.4)	5 (0.0)	< 0.0001
Atrial fibrillation	798 (9.2)	363 (2.2)	< 0.0001
Ventricular tachycardia/Torsade de pointes	92 (1.1)	22 (0.1)	< 0.0001
Supra ventricular tachycardia	61 (0.7)	35 (0.2)	< 0.0001
Atrioventricular block II/III	75 (0.9)	33 (0.2)	< 0.0001
Sick sinus syndrome	52 (0.6)	30 (0.2)	< 0.0001

Description of age, gender, incidence period and of relevant comorbidities prior to diagnosis in all individuals with myocarditis in Sweden from 2000 to 2014, as well as in their age and gender matched controls.

**Figure 1:**
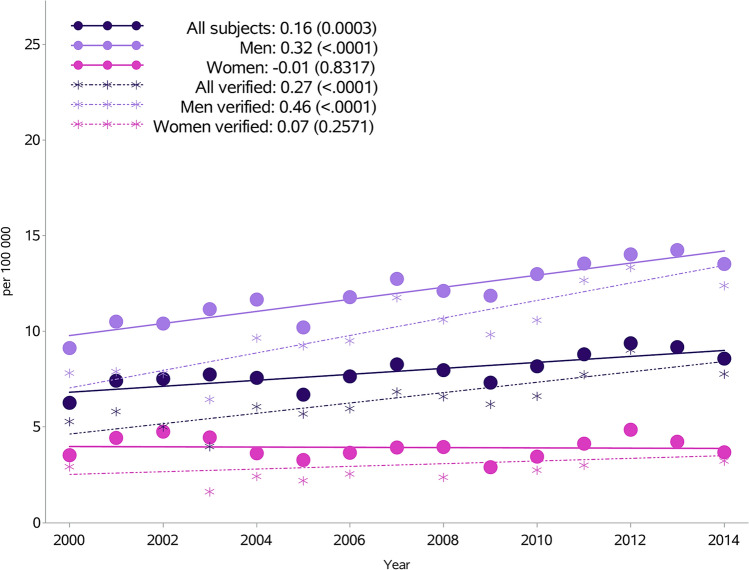
Incidence of myocarditis per 100,000 inhabitants in Sweden from 2000 to 2014 by sex, with or without validation in a subpopulation with a diagnosis of myocarditis validated from 2000 to 2014.

**Figure 2:**
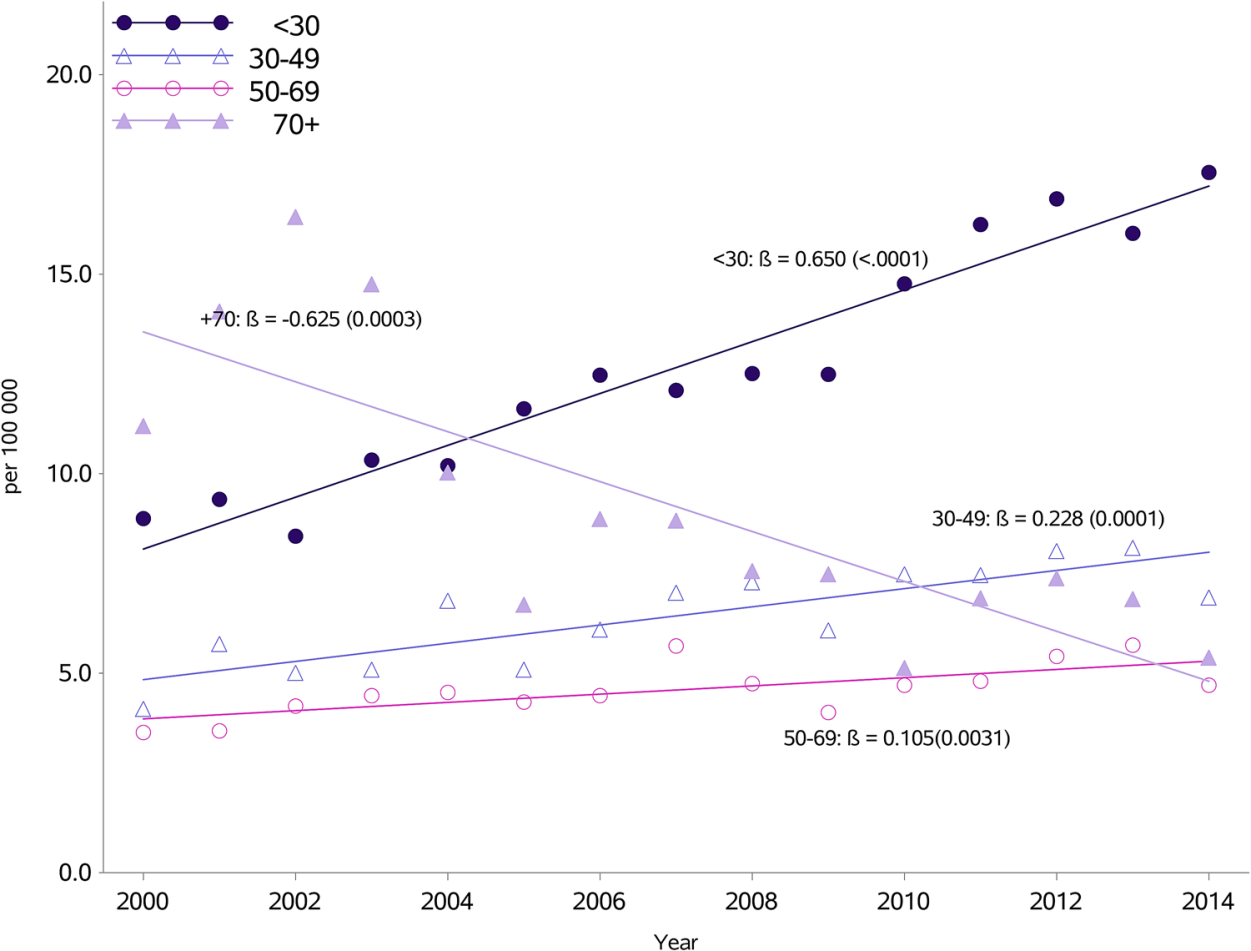
Incidence of myocarditis per 100,000 inhabitants in Sweden from 2000 to 2014 by age.

The overall incidence rate per 100,000 inhabitants increased from 6.3 in 2000 to 8.6 in 2014 (Fig. 1, Supplementary Table S1). However, while the standardized incidence in men has risen from 9.1 in 2000 to 13.5 in 2014 per 100,000, there was little change in women. When further subdivided by age, the most marked rise was in persons aged < 30 years (p < 0.0001) and 30–49 years (p < 0.0001). There was a slight increase in those aged 50–69 years (p = 0.003), whereas a decrease was seen in the age group ≥ 70 years (p = 0.0003) (Fig. 2). Rates in men aged < 50 years nearly doubled from 9.8 in 2000 to 18.6 per 100,000 in 2014 (p for trend < 0.0001), and similarly for women aged < 50 years, although at a much lower level, from 2.0 to 3.6 per 100,000 (p for trend < 0.0001) (Supplementary Table S2). There were downward trends in men and women ≥ 50 years.

In the subpopulation with a hospital diagnosis of myocarditis, validated through a review of medical case records, a similar rise in incidence was seen from 2000 to 2014 (from 5.3 to 7.8 per 100,000 inhabitants). The increasing incidence for men was from 7.8 in 2000 to 12.4 in 2014 and 2.9 in 2000 to 3.2 in 2014 in women (Fig. 1, Supplementary Table S1).

### Diagnoses of heart failure or dilated cardiomyopathy during follow-up

**Figure 3:**
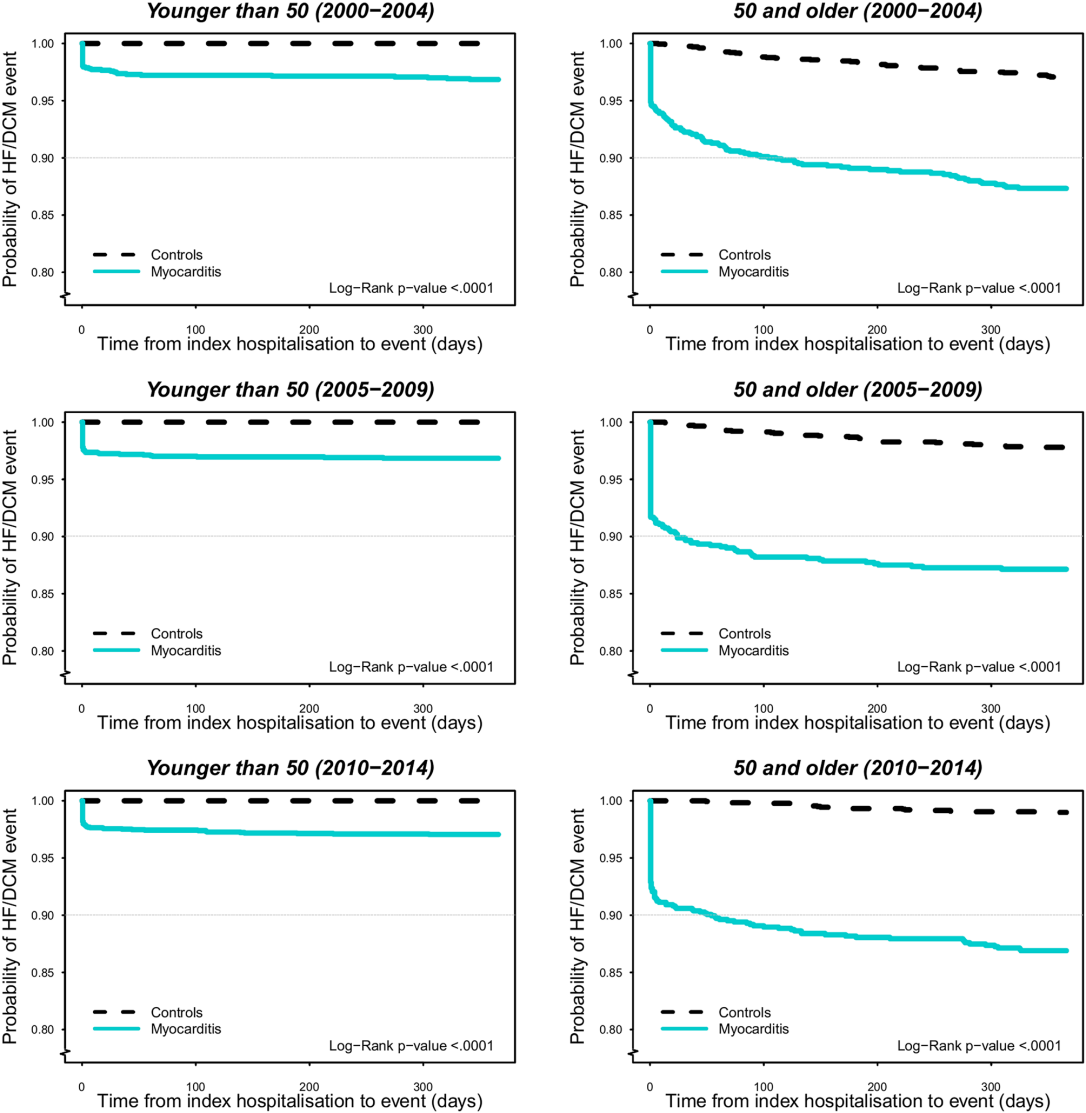
Probability of event heart failure/dilated cardiomyopathy 1 year after hospitalization from 2000 to 2014 stratified by age: < 50 and ≥ 50 years.

From the index hospitalization, 6.4% were newly diagnosed with either HF or DCM during the 1-year follow up while 9.3% developed HF/DCM during the entire study period (Table 2). Compared with those < 50 years old, a significantly higher incidence rate of HF/DCM was observed in those ≥ 50 years with a fourfold higher incidence (3.1% vs. 12.2%) during the first year after the index hospitalization, and a fivefold higher incidence (3.6% vs. 19.2%) during the entire study period. The risk for the development of HF/DCM was highest in the immediate post-discharge period after index hospitalization, especially in those ≥ 50 years (Fig. 3). Regardless of age and follow-up duration, the incidences of HF/DCM were much higher in individuals with myocarditis compared with the reference population.

**Table 2:** Hospitalizations for heart failure/cardiomyopathy, and deaths during study period.

	1-year follow-up		Whole study period	
	Case *n* (%)	Control *n* (%)	Case *n* (%)	Control *n* (%)
**HF/DCM**				
Whole period	554 (6.4)	115 (0.7)	805 (9.3)	644 (3.9)
2000–2004	181 (7.1)	59 (1.2)	322 (12.6)	394 (8.2)
2005–2009	178 (6.5)	38 (0.7)	255 (9.3)	191 (3.6)
2010–2014	195 (5.8)	18 (0.3)	228 (6.8)	60 (0.9)
** < 50 years**				
Whole period	169 (3.1)	0	198 (3.6)	26 (0.2)
2000–2004	43 (3.1)	0	56 (4.1)	16 (0.6)
2005–2009	56 (3.1)	0	64 (3.6)	8 (0.2)
2010–2014	70 (2.9)	0	78 (3.3)	2 (0.0)
** ≥ 50 years**				
Whole period	385 (12.2)	115 (2.1)	607 (19.2)	618 (11.0)
2000–2004	138 (11.6)	59 (2.9)	266 (22.3)	377 (18.3)
2005–2009	122 (12.5)	38 (2.2)	191 (19.5)	183 (10.4)
2010–2014	125 (12.7)	18 (1.0)	150 (15.2)	58 (3.2)
**Mortality**				
Whole period	705 (8.1)	330 (2.0)	1694 (19.5)	2067 (12.4)
2000–2004	350 (13.7)	191 (4.0)	936 (36.6)	1305 (27.3)
2005–2009	188 (6.8)	87 (1.6)	468 (17.0)	571 (10.8)
2010–2014	167 (5.0)	52 (0.8)	290 (8.6)	191 (2.9)
** < 50 years**				
Whole period	50 (0.9)	23 (0.2)	115 (2.1)	145 (1.3)
2000–2004	14 (1.0)	10 (0.4)	46 (3.4)	82 (3.0)
2005–2009	14 (0.8)	8 (0.2)	30 (1.7)	47 (1.3)
2010–2014	22 (0.9)	5 (0.1)	39 (1.6)	16 (0.3)
** ≥ 50 years**				
Whole period	655 (20.8)	307 (5.5)	1579 (50.1)	1922 (34.2)
2000–2004	336 (28.2)	181 (8.8)	890 (74.7)	1223 (59.4)
2005–2009	174 (17.8)	79 (4.5)	438 (44.8)	524 (29.8)
2010–2014	145 (14.7)	47 (2.6)	251 (25.5)	175 (9.7)

*HF* heart failure, *DCM* dilated cardiomyopathy.

### One-year mortality: trend over time and cause of death

**Figure 4:**
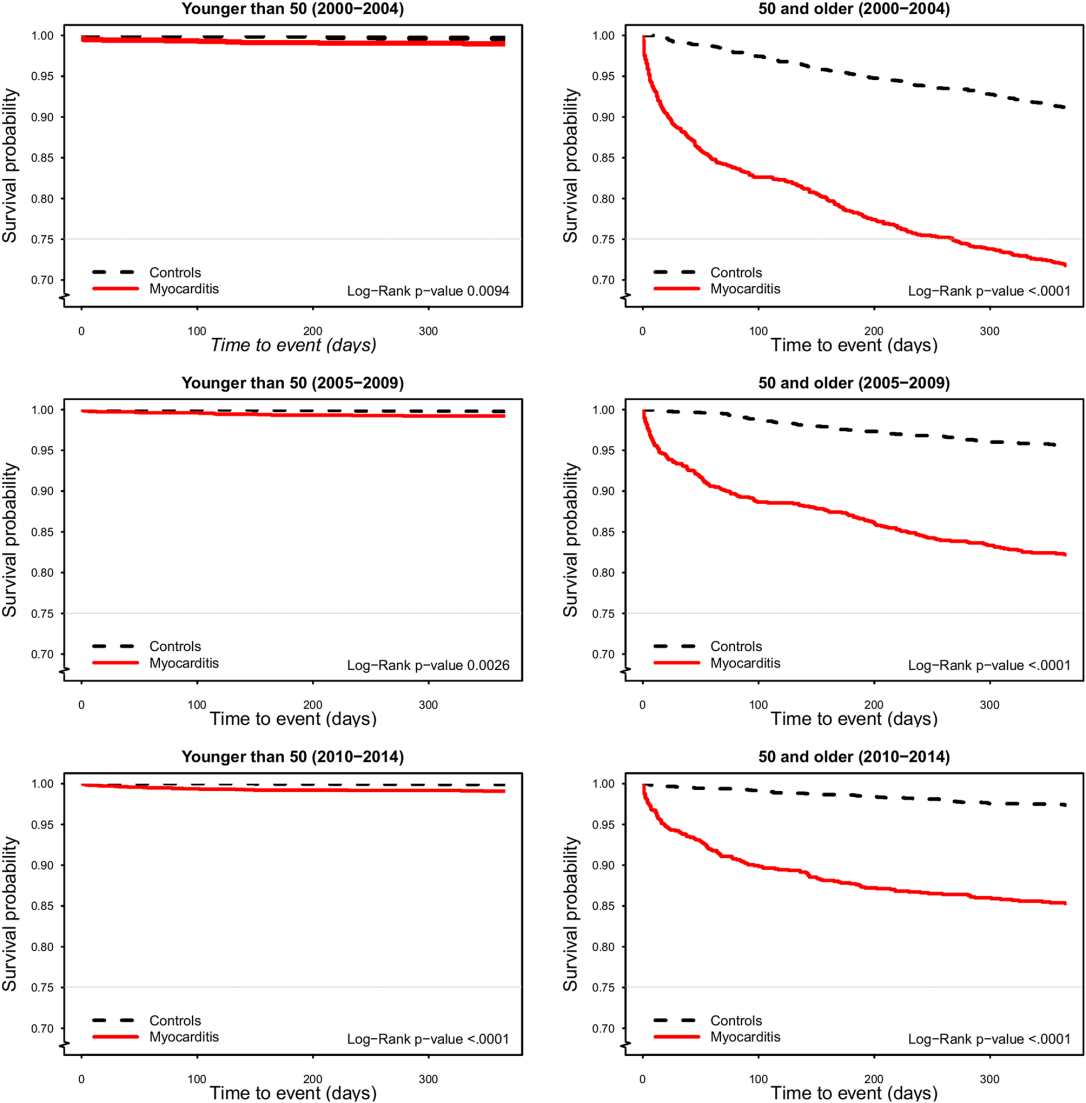
1-year mortality from 2000 to 2014 stratified by age: < 50 and ≥ 50 years.

During the first year after hospitalization, 705 (8.1%) of the subjects with myocarditis died and 330 (2.0%) in the reference population; during the entire study period, 1694 (19.5%) of the patients with myocarditis and 2067 (12.4%) in the reference population died (Table 2). When stratified by age, mortality was much higher in patients aged ≥ 50 years, of whom 20.8% died in the first year after hospitalization compared with only 0.9% in those < 50 (Table 2). Still, when comparing patients to controls, HRs for 1-year mortality were not conspicuously different across age groups in models adjusted for age, sex and period of the study. In patients < 50 years 1-year mortality did not decrease significantly from the first period (2000–2004) to the last (2010–2014), whereas there was a marked and significant decrease in patients ≥ 50 years (p < 0.001) (Supplementary Table S3). Moreover, the risk of death was highest in the immediate post-discharge period after index hospitalization, especially in those ≥ 50 years (Fig. 4). For cause of death, cardiovascular causes contributed to about 50% of the deaths in the myocarditis group. However, this percentage decreased from 70 in 2000 to 50% in 2014, whereas in the reference population < 50% of the deaths were due to cardiovascular causes, decreasing from 44 to 29% between 2000 and 2014.

## Discussion

In this population-based cohort, established on hospitalization for myocarditis according to the Swedish NPR database, the following major findings were observed: (1) A slightly increased incidence of myocarditis per 100,000 inhabitants (from 6.3 in 2000 to 8.6 in 2014); (2) a decline in 1-year mortality in patients with myocarditis during 2000–2014; (3) an observed reduction in the mortality during the study period in patients ≥ 50 years of age.

The incidence of myocarditis in the general population is at present unknown. Studies have shown suspected myocarditis in 3.5–5% of patients during outbreaks of Coxsackievirus infection^7,13^. The “golden standard” for diagnosis of myocarditis is endomyocardial biopsy. However, this is infrequently used, and has limited sensitivity^4,14^. Autopsy studies have shown estimates of the prevalence of myocarditis to be 2–12% in those who died suddenly^15–17^. Other studies reported myocarditis as a cause of initially unexplained DCM in 9%^18^ and 10% in unexplained HF^9^. Due to evolving diagnostic criteria and differences in the conceptual view and interpretation of myocarditis within the medical community, definitions associated with myocarditis have changed over the last decades.

Our data showed a slight increment of incidence of clinical suspected myocarditis during a period of 15 years. Despite that endomyocardial biopsy is the diagnostic gold standard, it is seldom used in daily clinical practice, partly because of its low sensitivity^1^, but also because myocarditis is often a self-limiting and mostly benign condition for which an invasive procedure is not warranted. The latest Expert Consensus Document^19^ recommends endomyocardial biopsy in a limited number of clinical scenarios, including hemodynamic compromise, patients with life-threatening arrhythmia. Therefore, noninvasive examinations and biomarkers are still essential for the recognition of myocarditis in clinical practice. During the past few decades, the application of biomarkers has notably improved. Cardiac troponins are more sensitive to myocyte injury than creatinine kinase in patients with clinically suspected myocarditis^20,21^. In Sweden, troponin T assay was introduced in 1997, which certainly contributed to better detection of myocarditis. However, the increased incidence of myocarditis cannot be sole explained by improvements in biomarker sensitivity, as the increasing trend could be seen already from when coverage for the NPR became nationwide in 1987 (data not shown). The increase might be attributable to other reasons, such as greater awareness among physicians of the myocarditis diagnosis, but also a possible real increase in myocarditis due to a rise in viral infections. Thus, our data may well indicate a true increase in myocarditis over the past decade.

Little information is available about the development of HF/DCM in patients with myocarditis. In our previous studies^22,23^ we hypothesized that an increased incidence of myocarditis might at least partly account for the rise in the incidence of HF in the younger (< 50 years) sector of the population. Moreover, some studies reported persistent cardiac dysfunction in about 25% of cases and either death or end-stage DCM in 12–25%^1–6,24,25^. In the current study, we found that 6.4% of the patients with myocarditis developed incident HF/DCM within 1 year after the index hospitalization and 9.3% throughout the study period. The incident rates of HF/DCM were higher from 2000 to 2004, with 7.1% developing these conditions within 1 year after index hospitalization, compared with 5.8% in the last period. For patients ≥ 50 years of age, the incidence of HF/DCM was much higher, with 12% during the first year after the index hospitalization and approximately 19% during long-term follow-up, which is close to previously reported rates^1–6,24,25^. Of note, we observed a steadily decreased incidence of HF/DCM over time, which might be attributable to improved diagnosis and medical care of myocarditis, as well as of hypertension and ischemic heart disease which also causes HF. The highest risk of both HF/DCM and death occurred in the immediate post-discharge period after index hospitalization, in particular in those ≥ 50 years, suggesting that there is great potential for further improvement of acute care of myocarditis and subsequent follow-up.

Despite higher mortality in patients with myocarditis than in the reference population, mortality continued to decrease in the past decade (2000–2014). The declining trend in 1-year mortality in patients with myocarditis is probably multifactorial, partly attributable to earlier recognition of myocarditis and improved acute management, including HF treatment. Even though we noted a similar trend in mortality in the reference population, the fall was steeper in those diagnosed with myocarditis. The higher 1-year mortality in the myocarditis group, as compared with the reference population, indicates that myocarditis is not an entirely benign condition. Identifying the risk factors for cardiovascular death in patients with myocarditis is imperative and will be the focus of our future research.

### Limitations and strengths

The main strengths of this study are access to data from practically all persons in Sweden and that the study covers an extended period. In Sweden, patients with suspected myocarditis based on symptoms and objective signs of cardiac dysfunction are routinely hospitalized. All hospitalized cases of myocarditis in Sweden during the study period are thus included in our database.

Limitations include that the study was conducted using data collected for administrative purposes and not for research. Although our review of 507 patient records showed that 83% were considered to be correctly diagnosed, individual validation of all cases identified on a national basis in our study was not possible. Still, we reviewed all case records of patients hospitalized with myocarditis at the Sahlgrenska University Hospital in Gothenburg, a conglomerate of three hospitals at different locations in the city including a tertiary care centre with access to advanced diagnostic tools including myocardial biopsy and imaging when indicated. In this subpopulation, we showed a similar trend with an increasing incidence as in the overall cohort, however, because there were no exact criteria for diagnosis, our findings with respect to trends must be regarded as tentative.

Still, in this study we performed hospital records in order to verify the myocarditis diagnoses at the Sahlgrenska University Hospital in Gothenburg—a conglomerate of three hospitals at different locations in the city—every fifth year and records for all patients with a diagnosis of myocarditis. In this diagnosis-validated subpopulation, we showed a similar trend with an increasing incidence as in the overall cohort.

In conclusion, by having access to the Swedish national databases (for hospitalization, cause of death and the general population), we could show a potentially increasing trend in the incidence of myocarditis over time but also a declining trend in both development of HF/DCM and mortality in these patients.

## Supplementary Information

Supplementary Tables.

## Data Availability

The data underlying this article will be shared on reasonable request to the corresponding author.

## Abbreviations

CI: Confidence interval
CVD: Cardiovascular disease
DCM: Dilated cardiomyopathy
HF: Heart failure
HR: Hazard ratio
ICD: International Classification of Diseases
NDR: Swedish Cause of Death Register
NPR: Swedish National Patient Register
