# Supplementary material for: Trends in myocarditis incidence, complications and mortality in Sweden from 2000 to 2014
Source: Sci Rep. 2022 Feb 2;12:1810. doi: 10.1038/s41598-022-05951-z (PMC8810766; doi:10.1038/s41598-022-05951-z)
Supplement: Supplementary file 1 — Supplementary Tables. [file 41598_2022_5951_MOESM1_ESM.docx]

**Supplementary Table S1: Incidence rate of myocarditis by sex. Crude rates, overall incidence rate per 100 000 inhabitants and incidence rates per 100 000 inhabitants I the group with verified diagnosis.**

|  | ***Crude rates*** | | | ***Standardized rates per 100 000 inhabitants*** | | | ***Standardized rates per 100 000 inhabitants by verified diagnosis*** | | |
| --- | --- | --- | --- | --- | --- | --- | --- | --- | --- |
| ***Year*** | ***All*** | ***Men*** | ***Women*** | ***All*** | ***Men*** | ***Women*** | ***All*** | ***Men*** | ***Women*** |
| 2000 | 435 (3.5) | 310 (3.5) | 125 (3.6) | 6.3 | 9.1 | 3.5 | 5.3 | 7.8 | 2.9 |
| 2001 | 517 (4.2) | 359 (4.0) | 158 (4.6) | 7.4 | 10.5 | 4.4 | 5.8 | 7.9 | 4.4 |
| 2002 | 527 (4.3) | 357 (4.0) | 170 (4.9) | 7.5 | 10.4 | 4.8 | 5.0 | 7.8 |  |
| 2003 | 545 (4.4) | 385 (4.3) | 160 (4.7) | 7.7 | 11.2 | 4.5 | 4.0 | 6.4 | 1.6 |
| 2004 | 536 (4.3) | 405 (4.5) | 131 (3.8) | 7.6 | 11.7 | 3.6 | 6.1 | 9.7 | 2.4 |
| 2005 | 476 (3.8) | 357 (4.0) | 119 (3.5) | 6.7 | 10.2 | 3.3 | 5.7 | 9.2 | 2.2 |
| 2006 | 549 (4.4) | 416 (4.6) | 133 (3.9) | 7.6 | 11.8 | 3.6 | 6.0 | 9.5 | 2.6 |
| 2007 | 600 (4.8) | 455 (5.1) | 145 (4.2) | 8.3 | 12.7 | 3.9 | 6.8 | 11.8 |  |
| 2008 | 585 (4.7) | 438 (4.9) | 147 (4.3) | 8.0 | 12.1 | 4.0 | 6.6 | 10.6 | 2.4 |
| 2009 | 544 (4.4) | 435 (4.9) | 109 (3.2) | 7.3 | 11.9 | 2.9 | 6.2 | 9.8 | 2.9 |
| 2010 | 612 (4.9) | 481 (5.4) | 131 (3.8) | 8.2 | 13.0 | 3.5 | 6.6 | 10.6 | 2.8 |
| 2011 | 665 (5.4) | 507 (5.7) | 158 (4.6) | 8.8 | 13.6 | 4.1 | 7.7 | 12.7 | 3.0 |
| 2012 | 716 (5.8) | 529 (5.9) | 187 (5.4) | 9.4 | 14.0 | 4.9 | 9.0 | 13.3 | 4.9 |
| 2013 | 707 (5.7) | 543 (6.1) | 164 (4.8) | 9.2 | 14.3 | 4.2 | 9.2 | 14.3 | 4.2 |
| 2014 | 665 (5.4) | 521 (5.8) | 144 (4.2) | 8.6 | 13.5 | 3.7 | 7.8 | 12.4 | 3.2 |

**Supplementary Table S2: Incidence rate by age and sex**

|  | ***Crude rates*** | | | ***Standardized rates per 100 000* inhabitants** | | |
| --- | --- | --- | --- | --- | --- | --- |
| ***Age <50 years*** | ***All*** | ***Men*** | ***Women*** | ***All*** | ***Men*** | ***Women*** |
| 2000 | 234 (3.0) | 196 (3.1) | 38 (2.7) | 5.9 | 9.8 | 2.0 |
| 2001 | 281 (3.6) | 236 (3.7) | 45 (3.2) | 7.1 | 11.7 | 2.3 |
| 2002 | 250 (3.2) | 215 (3.4) | 35 (2.5) | 6.3 | 10.7 | 1.8 |
| 2003 | 281 (3.6) | 246 (3.9) | 35 (2.5) | 7.1 | 12.2 | 1.8 |
| 2004 | 323 (4.2) | 282 (4.4) | 41 (3.0) | 8.1 | 13.9 | 2.1 |
| 2005 | 305 (3.9) | 261 (4.1) | 44 (3.2) | 7.6 | 12.8 | 2.2 |
| 2006 | 348 (4.5) | 298 (4.7) | 50 (3.6) | 8.6 | 14.4 | 2.5 |
| 2007 | 370 (4.8) | 318 (5.0) | 52 (3.7) | 9.0 | 15.2 | 2.6 |
| 2008 | 389 (5.0) | 330 (5.2) | 59 (4.3) | 9.4 | 15.6 | 2.9 |
| 2009 | 364 (4.7) | 327 (5.1) | 37 (2.7) | 8.7 | 15.3 | 1.8 |
| 2010 | 442 (5.7) | 377 (5.9) | 65 (4.7) | 10.4 | 17.4 | 3.1 |
| 2011 | 470 (6.1) | 393 (6.2) | 77 (5.6) | 11.0 | 18.1 | 3.7 |
| 2012 | 498 (6.4) | 411 (6.5) | 87 (6.3) | 11.7 | 18.9 | 4.2 |
| 2013 | 486 (6.3) | 406 (6.4) | 80 (5.8) | 11.4 | 18.6 | 3.8 |
| 2014 | 483 (6.2) | 407 (6.4) | 76 (5.5) | 11.3 | 18.6 | 3.6 |
| ***Age ≥50 years*** |  |  |  |  |  |  |
| 2000 | 201 (4.3) | 114 (4.4) | 87 (4.2) | 6.3 | 7.6 | 5.1 |
| 2001 | 236 (5.1) | 123 (4.8) | 113 (5.5) | 7.3 | 8.2 | 6.5 |
| 2002 | 277 (6.0) | 142 (5.5) | 135 (6.6) | 8.5 | 9.3 | 7.8 |
| 2003 | 264 (5.7) | 139 (5.4) | 125 (6.1) | 8.0 | 9.0 | 7.1 |
| 2004 | 213 (4.6) | 123 (4.8) | 90 (4.4) | 6.4 | 7.9 | 5.1 |
| 2005 | 171 (3.7) | 96 (3.7) | 75 (3.7) | 5.1 | 6.1 | 4.2 |
| 2006 | 201 (4.3) | 118 (4.6) | 83 (4.0) | 5.9 | 7.4 | 4.6 |
| 2007 | 230 (5.0) | 137 (5.3) | 93 (4.5) | 6.7 | 8.5 | 5.2 |
| 2008 | 196 (4.2) | 108 (4.2) | 88 (4.3) | 5.7 | 6.6 | 4.9 |
| 2009 | 180 (3.9) | 108 (4.2) | 72 (3.5) | 5.2 | 6.5 | 3.9 |
| 2010 | 170 (3.7) | 104 (4.0) | 66 (3.2) | 4.8 | 6.2 | 3.6 |
| 2011 | 195 (4.2) | 114 (4.4) | 81 (4.0) | 5.5 | 6.8 | 4.4 |
| 2012 | 218 (4.7) | 118 (4.6) | 100 (4.9) | 6.1 | 6.9 | 5.4 |
| 2013 | 221 (4.8) | 137 (5.3) | 84 (4.1) | 6.1 | 7.9 | 4.5 |
| 2014 | 182 (3.9) | 114 (4.4) | 68 (3.3) | 5.0 | 6.5 | 3.6 |

**Supplementary Table S3.** Hazard ratios for 1-year mortality in cases vs. controls in multivariable analyses

|  | ***All*** | |  | ***Men*** | |  | ***Women*** | |
| --- | --- | --- | --- | --- | --- | --- | --- | --- |
| **<30 years** | ***HR (95%)*** | ***p-value*** |  | ***HR (95%)*** | ***P-value*** |  | ***HR (95%)*** | ***P-value*** |
| ***Myocarditis, case/control*** | 4.00 (1.37-11.70) | 0.0114 |  | 2.80 (0.89-8.81) | 0.0790 |  | NA |  |
| *Age* | 0.99 (0.87-1.13) | 0.9212 |  | 1.05 (0.91-1.21) | 0.5516 |  | 0.78 (0.52-1.16) | 0.2147 |
| *Sex (male)* | 0.54 (0.15-1.92) | 0.3425 |  |  |  |  |  |  |
| *Period (2005-2009)* | 0.91 (0.24-3.40) | 0.8872 |  | 0.96 (0.21-4.30) | 0.9576 |  | 0.72 (0.05-11.55) | 0.8169 |
| *Period (2010-2014)* | 0.74 (0.21 -2.63) | 0.6428 |  | 0.87 (0.21-3.64) | 0.8469 |  | 0.41 (0.03-5.55) | 0.5280 |
| ***30-<50 years*** |  |  |  |  |  |  |  |  |
| ***Myocarditis, case/control*** | 4.48 (2.57-7.82) | <.0001 |  | 3.39 (1.71-6.74) | 0.0005 |  | 7.32 (2.72-19.72) | <.0001 |
| *Age* | 1.13 (1.08-1.19) | <.0001 |  | 1.13 (1.06-1.20) | 0.0001 |  | 1.13 (1.05-1.23) | 0.0025 |
| *Sex (male)* | 0.40 (0.23-0.67) | 0.0006 |  |  |  |  |  |  |
| *Period (2005-2009)* | 0.67 (0.35-1.28) | 0.2257 |  | 0.75 (0.30-1.84) | 0.5255 |  | 0.58 (0.23-1.47) | 0.2520 |
| *Period (2010-2014)* | 0.65 (0.35-1.20) | 0.1689 |  | 1.07 (0.49-2.37) | 0.8591 |  | 0.27 (0.09-0.80) | 0.0179 |
| ***50-<70 years*** |  |  |  |  |  |  |  |  |
| ***Myocarditis, case/control*** | 4.57 (3.31-6.31) | <.0001 |  | 4.06 (2.73-6.05) | <.0001 |  | 5.66 (3.25-9.85) | <.0001 |
| *Age* | 1.05 (1.02-1.08) | 0.0006 |  | 1.08 (1.04-1.11) | <.0001 |  | 1.00 (0.96-1.05) | 0.9421 |
| *Sex (male)* | 1.07 (0.78-1.46) | 0.6686 |  |  |  |  |  |  |
| *Period (2005-2009)* | 0.63 (0.45-0.89) | 0.0096 |  | 0.62 (0.40-0.95) | 0.0286 |  | 0.63 (0.35-1.13) | 0.1245 |
| *Period (2010-2014)* | 0.44 (0.30-0.63) | <.0001 |  | 0.38 (0.24-0.62) | <.0001 |  | 0.52 (0.29-0.95) | 0.0342 |
| ***≥70 years*** |  |  |  |  |  |  |  |  |
| ***Myocarditis, case/control*** | 3.93 (3.39-4.57) | <.0001 |  | 4.32 (3.51-5.32) | <.0001 |  | 3.55 (2.86-4.41) | <.0001 |
| *Age* | 1.09 (1.07-1.10) | <.0001 |  | 1.09 (1.07-1.10) | <.0001 |  | 1.09 (1.07-1.10) | <.0001 |
| *Sex (male)* | 1.41 (1.22-1.62) | <.0001 |  |  |  |  |  |  |
| *Period (2005-2009)* | 0.76 (0.64-0.90) | 0.0012 |  | 0.76 (0.60-0.95) | 0.0183 |  | 0.76 (0.60-0.97) | 0.0290 |
| *Period (2010-2014)* | 0.63 (0.53-0.76) | <.0001 |  | 0.64 (0.50-0.83) | 0.0006 |  | 0.63 (0.47-0.83) | 0.0009 |
